# Fulminant Guillain–Barré Syndrome Post Hemorrhagic Stroke: Two Case Reports

**DOI:** 10.3390/neurolint13020019

**Published:** 2021-05-06

**Authors:** Sameeh Abdulmana, Naif Al-Zahrani, Yahya Sharahely, Shahid Bashir, Talal M. Al-Harbi

**Affiliations:** 1 Neuroscience Centre, Neurology Department, King Fahad Specialist Hospital-Dammam, Dammam 31444, Saudi Arabia; Sameeh.Omar@kfsh.med.sa (S.A.); Naif.Zahrani@kfsh.med.sa (N.A.-Z.); sbashir10@gmail.com (S.B.); 2 Neurology Division, Medical Department, Dammam Medical Complex, Dammam 31444, Saudi Arabia; Ysharahely@moh.gov.sa

**Keywords:** Guillain–Barré syndrome, Guillain–Barré following stroke, intracranial hemorrhage, hemorrhagic stroke complicated by radiculopathy

## Abstract

Guillain–Barré syndrome (GBS) is an acute, immune-mediated inflammatory peripheral polyneuropathy characterized by ascending paralysis. Most GBS cases follow gastrointestinal or chest infections. Some patients have been reported either following or concomitant with head trauma, neurosurgical procedures, and rarely hemorrhagic stroke. The exact pathogenesis is not entirely understood. However, blood–brain barrier damage may play an essential role in triggering the autoimmune activation that leads to post-stroke GBS. Here, we present two cases of fulminant GBS following hemorrhagic stroke to remind clinicians to be aware of this rare treatable complication if a stroke patient develops unexplainable flaccid paralysis with or without respiratory distress.

## 1. Introduction

Guillain, Barré, and Strohl first reported Guillain–Barré syndrome (GBS), or acute inflammatory demyelinating polyradiculoneuropathy, in 1916 [1]. It is characterized by acute ascending progressive weakness, areflexia, and dysautonomia [2]. Clinically, the incidence of GBS is rare, at 0.8–1.9/100,000 people/year [1]. Several subtypes of GBS are well recognized, including acute inflammatory demyelinating polyradiculoneuropathy (AIDP), acute motor axonal neuropathy (AMAN), acute motor-sensory axonal neuropathy (AMSAN), and Miller Fisher syndrome (MFS). 

GBS is thought to result from an immune response that leads to acute polyneuropathy. It is often triggered by an antecedent infection. Two-thirds of patients report symptoms suggesting an initial respiratory or gastrointestinal disease [3]. A minority of patients develop GBS following a vaccine, bone marrow transplant, neurosurgery, or head trauma. However, in the last few years, growing reports about the concurrence of GBS with unusual events like intracerebral hemorrhage (ICH) have increased with unfavorable prognosis due to cardiovascular autonomic instability [4]. 

Here, we report two cases of patients who were diagnosed with GBS following hemorrhagic stroke. The first patient developed GBS after endovascular mechanical thrombectomy for acute ischemic stroke complicated by a hemorrhagic transformation. In comparison, the second patient developed GBS after hematoma evacuation.

## 2. Case Presentation

### 2.1. Case 1

The patient was a 74-year-old right-handed woman. She was known to have type 2 diabetes (DM), hypertension (HTN), and dyslipidemia. She presented to the emergency department (ED) after six hours of left-sided weakness and dysarthria in February 2019. Her neurological examination was remarkable for dysarthria, as she was able to obey simple commands, and showed right gaze preference, left facial (sparing the forehead) flaccid weakness (power 0/5), and an extensor plantar reflex. 

**Figure 1:**
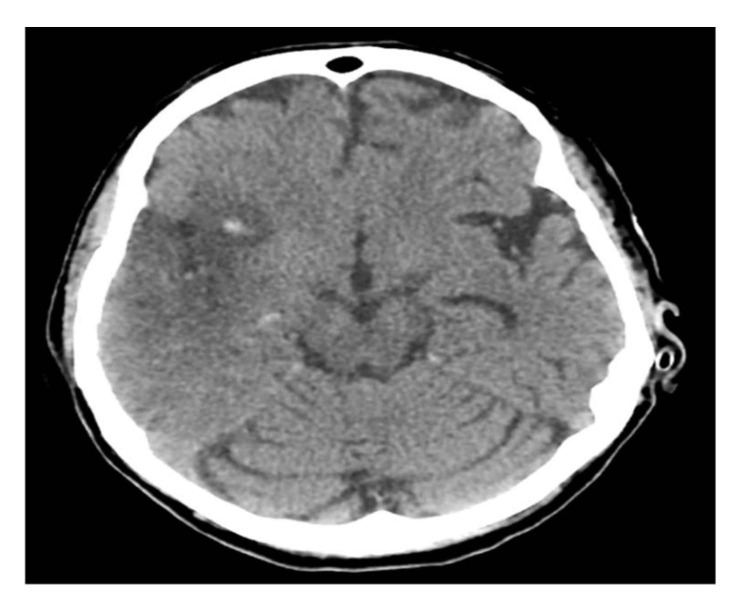
Brain CT scan.

A computerized brain tomography (CT) and CT arteriogram (CTA) showed acute right middle cerebral artery (MCA) ischemic infarction with complete occlusion of the suitable M1 and a partial filling defect in the distal M2 segment. She underwent endovascular thrombectomy with partial recanalization and was kept in an intensive care unit (ICU) for close monitoring. A follow-up CT brain showed interval development of hemorrhagic transformation (Figure 1). 

Three days later, she had moved to the ward in a stable condition, with minimal improvement in her left side weakness. On the fifth day of admission, she developed breathing difficulty and consciousness level deterioration. Her arterial blood gas (ABG) revealed high PaCo2 65 mm Hg (45–45 mm Hg), normal bicarbonate HCO3 23 mEq/L (22–26 mEq/L), low oxygen PO2 70 mm Hg (80–100 mm Hg), and low Ph 7.24 (7.35–7.45); subsequently, she required ICU admission, was intubated, and was connected to mechanical ventilation.

Her neurological examination at that time revealed absent brain stem reflexes with bilateral facial weakness. She had flaccid quadriplegia with areflexia. Her metabolic workup, including electrolytes level, renal, liver, and thyroid function, was normal. There was no clinical nor metabolic evidence of infection. Chest X-ray, blood culture, and urine culture were negative for any abnormalities. The brain MRI (Supplementary Figure S1) demonstrated an interval evolution of the known right MCA territories infarctions with a hemorrhagic transformation and no brain stem involvement. Because she was unconscious, electroencephalography (EEG) was done, which showed generalized diffuse slowing with no epileptiform activity. Based on her clinical progression, a diagnosis of GBS was considered.

Two weeks later, the nerve conduction study demonstrated absent motor responses of the common peroneal, tibial, median, and ulnar nerves and absent sensory responses from the sural, superficial peroneal, median, and ulnar nerves with stimulation of the four limbs (Supplementary Table S1). Moreover, the needle examination for the non-paralyzed side from the stroke, including right tibialis anterior, gastrocnemius, vastus medialis, first dorsal interosseous, biceps, triceps, and deltoid, showed diffuse abnormal spontaneous discharges in forms of positive sharp waves (PSWs) and fibrillation potentials, which were more abundant in the distal muscles with no voluntary motor units, which is highly compatible with AMSAN variant. The cerebral spinal fluid (CSF) analysis showed albuminocytological dissociation with a normal white blood cells (WBCs) of 1 and an extremely high protein of 3700 mg/L. More interestingly, her serum antiganglioside antibodies were positive for GM1 antibodies 95 IV (reference value: 0–50), GD1a 105 IV (reference value: 0–50), and GD1B 115 IV (reference value: 0–50), and negative for GQ1b 9 IV (reference value: 0–50). Therefore, a trial of IVIg 0.4 g/Kg per day for a total of 5 days was administered. Unfortunately, the patient remained clinically unresponsive and stayed on full ventilator support on tracheostomy for two months. In May 2019, she passed away due to severe dysautonomia. 

### 2.2. Case 2

A 61-year-old right-handed gentleman, known to have uncontrolled DM, and HTN, presented to ED with headache, impaired level of consciousness (LOC), and left-sided weakness. A brain CT (Supplementary Figure S2) showed an acute large right front-parietal ICH and a midline shift. There was no apparent underlying intracranial aneurysm or vascular malformation in the CTA.

He was intubated and underwent decompressive craniotomy for hematoma evacuation. The CT brain, post-operation, showed an interval reduction of the right frontoparietal intraparenchymal hemorrhage.

One week later, he became unresponsive with a GCS of 3/15 quadriplegic and areflexic. There was no new insult to the brain, but interval regression of intracranial bleeding was observed in his follow-up CT brain. 

His septic workup was unremarkable, and the EEG demonstrated generalized diffuse slowing. On the other hand, the spinal MRI (Supplementary Figure S3) showed diffuse contrast enhancement of the cauda equina roots. 

The nerve conduction study was done ten days later, demonstrating absent sensory, motor, and F responses from the upper and lower limbs’ nerves (Supplementary Table S2). Also, the needle examination showed diffuse PSWs and fibrillation potentials in the distal limb muscles (both sides). Furthermore, the CSF analysis showed a WBC 10 (lymph predominant 96%) and high protein 1611 mg/L, and positive serum antiganglioside antibodies. He had been given an IVIg course with a total dose of 2 g per kg divided into five days. Within a month, an improvement in his consciousness level was observed. However, he remained with flaccid quadriplegia, having bulbar weakness and respiratory failure, on alternative pressure support and T-vent. After receiving ten sessions of plasma exchange, he gradually weaned off mechanical ventilator support. He was shifted to the floor in a relatively stable condition with no significant improvement in his weakness.

## 3. Discussion

We reported two patients with severe GBS following intracerebral hemorrhage (ICH) and acute ischemic stroke complicated with hemorrhagic transformation post endovascular therapy. Both patients developed hyperacute generalized weakness and areflexia associated with respiratory failure during their recovery stage following hemorrhagic stroke. Moreover, the CSF analysis of our two patients showed albuminocytological dissociation, supportive of polyradiculopathy. The brain MRI of both cases showed no evidence of abnormality in the brain stem. On the other hand, the second patient’s spinal MRI demonstrated roots contrast enhancement, which is also highly suggestive of GBS. For both patients, nerve conduction study (NCS) showed absent compound muscle action potentials (CMAPs), sensory nerve action potentials (SNAPs), and F wave latencies, indicating motor and sensory axonal injury, which are common electrophysiological features of AMSAN. Therefore, the GBS diagnosis in both patients accomplished the GBS diagnostic criteria published in 2014 [5]. 

Impaired consciousness and EEG slowing are not typical in GBS. Therefore, the differential diagnosis of these two patients may include Bickerstaff encephalitis (BBE). However, the absence of classical features of BBE, such as prodromal period of febrile illness, ophthalmoplegia, ataxia, and brain stem lesions in the MRI, in addition to negative anti Gq1b, are in favor of GBS diagnosis [6]. 

GBS’s pathogenesis following an ischemic stroke or ICH is not well understood; however, disruption of the blood–brain barrier due to hemorrhagic stroke serves as the sentinel event of flaccid quadriplegia, areflexia, hypotonia, and respiratory failure. 

Tan et al. [4] performed a sural nerve biopsy on a 44-year-old man who presented with severe GBS one week after a head injury. It showed profuse foamy macrophages in the endoneurium that phagocytosed the contents of endo-neural tubes, together with the presence of lymphocytes and severe axonal degeneration, which suggested that head trauma and surgery had elevated serum and CSF myelin basic protein levels, and subsequently immune system activation to produce phagocytosis of the myelin sheath, which caused demyelination. 

The initial insult plays an essential role in posttraumatic GBS pathogenesis due to disruption of the blood–brain barrier [7,8], leading to the release of immunologic components of neuronal debris (including myelin-associated proteins) into the blood, inducing the production of anti-myelin antibodies and subsequent neuropathy [9].

Another possible pathogenesis is the stress state after hemorrhage, in which disturbance of cellular humoral immunity is triggered by hemoglobin infiltration in the acute inflammation after brain injury [10,11,12]. Wu et al. (2016) reported a case of GBS following the hemorrhagic transformation of left middle cerebral artery infarction [10]. The impaired vascular autonomic function in GBS patients may be associated with rupture of cerebral micro-vessels, which is an adverse effect of immunoglobulin (hemodynamic changes, such as hypotension, hypertension, and tachycardia) that may be related to the ICH after GBS [13,14]. An incidence of 1–16.9% can occur with thromboembolic complications [13]. High blood viscosity, hyper coagulopathy, vasospasm, and autoimmune vasculitis are considered potential mechanisms. These changes in the blood vessel can cause vascular dysfunction as well. 

A striking feature in the first patient was absent brain stem reflexes mimicking brain death. There have been few reported cases about fulminant Guillain–Barré syndrome with absent brainstem reflexes [11] due to the virtually complete motor, sensory, and autonomic denervation indicating poor outcome.

Dysautonomia, including arrhythmia, hypertension, or hypotension, is associated with poor clinical outcomes and can occasionally turn fatal [12], as occurred in our first patient (Case 1). Concerning our reported cases, it is difficult to confirm whether there was a cause-and-effect relationship between these two entities or if it was merely a coincidence. 

We believe that further studies are required to understand better and clarify GBS’s association following hemorrhagic stroke. Most GBS patients have a good prognosis, yet death rates are still somewhere in the range of 4–5% [1]. GBS is still a life-threatening disorder, especially after a cerebral hemorrhagic injury; further research is required to develop therapeutics for GBS. Ventilatory issues, pneumonic inconveniences, and autonomic dysfunction are among the most common GBS complications that can increase the mortality rate [1]. The quality of life in such patients is impaired via disability, reduced myodynamia, pain, and fatigue [1]. 

## 4. Conclusions

These two case studies highlighted the importance of considering GBS in the differential diagnosis of patients with flaccid quadriparesis and respiratory failure after a hemorrhagic stroke. It may explain the clinical progression of these patients when early therapy could make a difference in their prognosis and outcome.

## Data Availability

The data is available on request from the corresponding author.

## Supplementary Materials

The following are available online at https://www.mdpi.com/article/10.3390/neurolint13020019/s1: Figure S1: Brain MRI (DWI), Figure S2: Brain CT, Figure S3: MRI lumbar spine T1 with contrast (axial view), Table S1: Electromyography and Nerve Conduction Studies report of Case 1, Table S2: Electromyography and Nerve Conduction Studies report of Case 2.
