# Supplementary material for: Fulminant Guillain–Barré Syndrome Post Hemorrhagic Stroke: Two Case Reports
Source: Neurol Int. 2021 May 6;13(2):190–4. doi: 10.3390/neurolint13020019 (PMC8162529; doi:10.3390/neurolint13020019)
Supplement: Supplementary file 1 [file neurolint-13-00019-s001.zip › neurolint-1082339-supplementary.pdf]

Supplementary Figure:

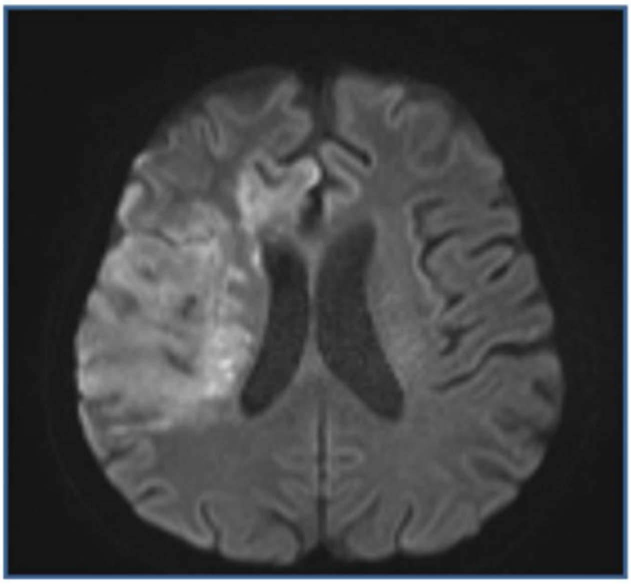

Figure S1. Brain MRI (DWI).

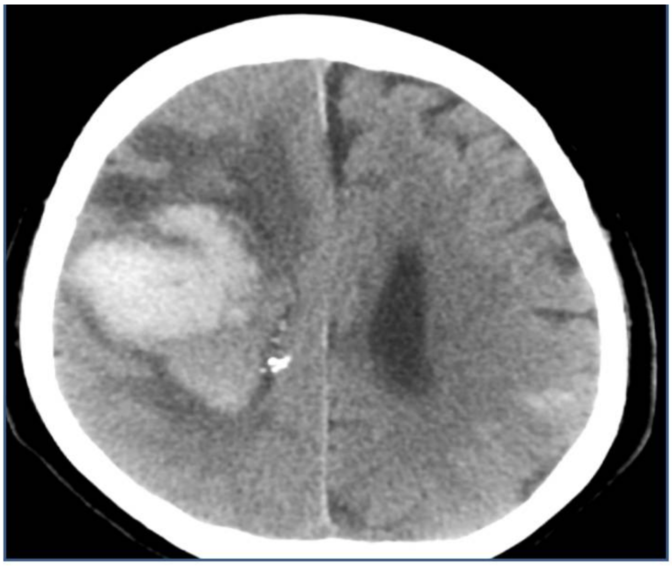

Figure S2. Brain CT.

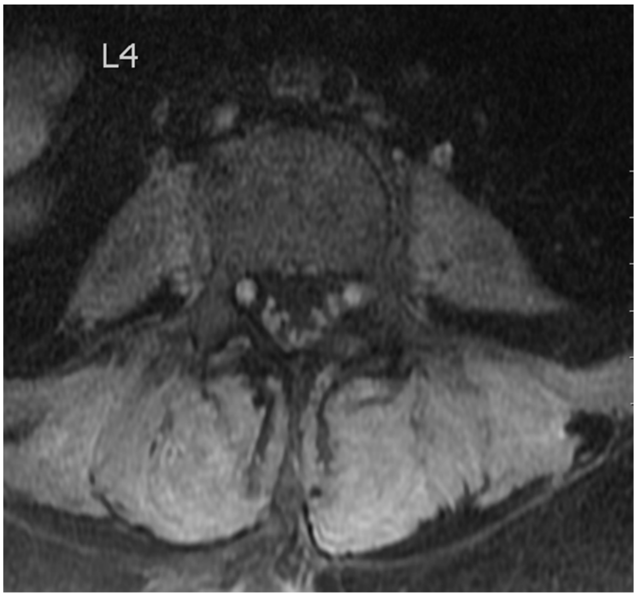

Figure S3. MRI lumbar spine T1 with contrast (axial view).

Supplementary Table:

Table S1. Electromyography (EMG) and nerve conduction studies report of Case 1.

Motor Nerve Conduction Studies (MNCS)

MNCS

Nerve	Lat	Amp	CV	F-M Lat	F Lat
	ms	mV	m/s	ms	ms
**Median Motor Right**					
Wrist - APB	--	--		--	--
Elbow-Wrist	--	--	--		
**Median Motor Left**					
Wrist – APB	--	--			--
Elbow-Wrist	--	--	--		
**Ulnar Motor Right**					
Wrist – ADM	--	--		--	--
Ab. Elbow-Wrist	--	--	--		
**Ulnar Motor Left**					
Wrist - ADM	--	--		--	--
Ab. Elbow-Wrist	--	--	--		
**Peroneal EDB Motor Right**					
Ankle – EDB	--	--		--	--
Bl. Knee-Ankle	--	--	--		
Ab. Knee-Bl. Knee	--	--	--		
**Peroneal EDB Motor Left**					
Ankle – EDB	--	--		--	--
Bl. Knee-Ankle	--	--	--		
Ab. Knee-Bl. Knee	--	--	--		
**Tibial Motor Right**					
Med. Mal – Abd Hal	--	--		--	--
Pop Fossa-Med. Mal	--	--	--		

**MNCS**					
**Tibial Motor Left**					
Med. Mal – Abd Hal	--	--		--	--
Pop Fossa-Med. Mal	--	--	--		

Lat: latency; Amp: amplitude; CV: conduction velocity; ms: millisecond; (m/s): meter/second;
APB: abductor pollicis brevis; ADM: abductor digiti minimi; EDB: extensor digitorum brevis;
Ab: above; Bl: below; Pop: popliteal; Abd Hal: abductor hallucis brevis; Med. Mal: medial malleolus.

Sensory Nerve Conduction Studies

**SNCS**			
Nerve	Peak Lat	Amp	CV
	ms	uV	m/s
**Median Sensory Right**			
Wrist - Dig II	--	--	--
**Median Sensory Right**			
Wrist - Dig II	--	--	--
**Ulnar Sensory Right**			
Dig V - Wrist	--	--	--
**Ulnar Sensory Left**			
Dig V - Wrist	--	--	--
**Radial Sensory Right**			
Forearm - Wrist	--	--	--
**Sural Sensory Right**			
Mid. lower leg - Ext Saph	--	--	--
**Sural Sensory Left**			
Mid. lower leg - Ext Saph	--	--	--

EMG Findings

	Insertion								
Muscle	Insertion Activity	Fib	PSW	Fasc	Amp	Dur	Poly	Stabil	Recruit
Right Deltoid Medial	Increased	++	+	-	No voluntary MUAP was recruited				
Right Biceps	Increased	++	++	+	No voluntary MUAP was recruited				
Left Biceps	Increased	++	++	+	No voluntary MUAP was recruited				
Left Triceps	Increased	++	++	+	No voluntary MUAP was recruited				
Right Triceps	Increased	++	++	-	No voluntary MUAP was recruited				
Right Vastus Medialis	Increased	++	++	-	No voluntary MUAP was recruited				
Left Vastus Lateralis	Increased	++	++	+	No voluntary MUAP was recruited				
Left Gastroc Med	Increased	++	++	+	No voluntary MUAP was recruited				
Right Gastroc Med	Increased	+++	+++	+	No voluntary MUAP was recruited				
Left Tibialis Anterior	Increased	+++	+++	+	No voluntary MUAP was recruited				
Right Tibialis Anterior	Increased	+++	+++	+	No voluntary MUAP was recruited				

Fib: fibrillation potentials; PSW: positive sharp waves; Fasc: fasciculations; Amp: amplitude; Dur: duration; Poly: polyphasic; Stabil: stability; - : Nil; +: 1+; ++: 2+; +++: 3+; Med: medial head; MUAP: motor unit action potentials.

Table S2: Electromyography (EMG) and Nerve Conduction Studies report of Case 2.

Motor Nerve Conduction Studies

Stim Site	NR	Onset (ms)	Peak (ms)	O-P Amp (mV)	Vel (m/s)
**Right Peroneal Motor (Ext Dig Brev)**					
Ankle	NR				
Poplt	NR				
**Right Median Motor (Abd Poll Brev)**					
Wrist	NR				
Elbow	NR				
**Right Tibial Motor (Abd Hall Brev)**					
Ankle	NR				
Knee	NR				
**Right Ulnar Motor (Abd Dig Min)**					
Wrist	NR				
A Elbow	NR				

Stim Site: stimulation Site; NR: not recorded; Vel: velocity; ms: millisecond; (m/s): meter/second;
O-P: onset to peak; Amp: amplitude; Ext. Dig Brev: extensor digitorum brevis; Poplit: popliteal;
Abd Poll Brev: abductor pollicis brevis; Abd Hall Brev: abductor hallucis brevis;
Abd Dig Min: abductor digiti minimi; A Elbow: above elbow.

F Wave Studies

NR	F-Lat (ms)	Lat Norm (ms)	L-R F-Lat (ms)	L-R Lat Norm
**Right Median (Abd Poll Brev)**				
NR				
**Right Ulnar (Abd Dig Min)**				
NR				

NR: not recorded; ms: millisecond; Lat: latency.

Sensory Nerve Conduction Studies

Stim Site	Onset (ms)	Peak (ms)	P-T Amp (µV)	Vel (m/s)
**Right Median Anti Sensory (2nd Digit)**				
Wrist	**NR**			
**Right Sural Anti Sensory (Lat Mall)**				
Calf	**NR**			
**Right Ulnar Anti Sensory (5th Digit)**				
Wrist	**NR**			

Lat: lateral malleolus.

EMG Findings

Muscle	Insertion<br>Insertion Activity	Fib	PSW	Fasc	Amp Dur Poly Stabil Recruit
Right Deltoid	Increased	++	+	-	No voluntary MUAP was recruited
Right Biceps	Increased	++	++	-	No voluntary MUAP was recruited
Left Biceps	Increased	++	++	-	No voluntary MUAP was recruited
Left Triceps	Increased	++	++	-	No voluntary MUAP was recruited
Right Triceps	Increased	++	++	-	No voluntary MUAP was recruited
Right Vastus Medialis	Increased	++	+	-	No voluntary MUAP was recruited
Left Gastroc Med	Increased	+++	+++	-	No voluntary MUAP was recruited
Right Gastroc Med	Increased	+++	+++	+	No voluntary MUAP was recruited
Left Tibialis anterior	Increased	+++	+++	+	No voluntary MUAP was recruited
Right Tibialis anterior	Increased	+++	+++	+	No voluntary MUAP was recruited

Fib: fibrillation potentials; PSW: positive sharp waves; Fasc: fasciculations; Amp: amplitude; Dur: duration; Poly: polyphasic; Stabil: stability; - : Nil; +: 1+; ++: 2+; +++: 3+; Med: medial head; MUAP: motor unit action potentials.
